# The effect of phosphorylated Akt inhibition on posterior capsule opacification in an ex vivo canine model

**Published:** 2010-10-29

**Authors:** Heather L. Chandler, Terah R. Webb, Curtis A. Barden, Mirunalni Thangavelu, Samuel K. Kulp, Ching-Shih Chen, Carmen M.H. Colitz

**Affiliations:** 1 College of Optometry, The Ohio State University, Columbus, OH; 2 College of Veterinary Medicine, The Ohio State University, Columbus, OH; 3 College of Pharmacy, The Ohio State University, Columbus, OH; 4 MedVet Medical Center for Pets, Worthington, OH

## Abstract

**Purpose:**

To evaluate whether inhibition of phosphorylated Akt (pAkt) would reduce or prevent posterior capsule opacification (PCO) in an ex vivo canine lens capsule model.

**Methods:**

Normal and cataractous lenses (n=6) were evaluated for pAkt via immunohistochemistry and immunoblotting. Primary cultures of lens epithelial cells (LEC) were exposed to ultraviolet light (UV) to induce pAkt. Cultures were then incubated in 0, 2.5, 5, or 10 µM (n=6) of a novel Akt inhibitor (AR-12) for either 8 or 24 h. Cultures were harvested and pAkt expression and telomerase activity examined by immunoblotting and telomeric repeat amplification protocol (TRAP)-enzyme linked immunosorbent assay (ELISA), respectively. Lens capsules were harvested post-sham cataract surgery and exposed to 0, 2.5, 5, 7.5, or 10 μM (n=8) of AR-12 for a total of 14 days treatment. Additional lens capsules (n=6) were exposed to 10 μM of AR-12 for 1 week followed by media alone for 1 week; or exposed to media alone for 1 week followed by 10 μM of AR-12 for 1 week. Histopathology and immunohistochemical staining were performed to evaluate PCO formation. Analysis of telomerase activity on the lens capsules was performed by TRAP-ELISA.

**Results:**

pAkt protein expression was increased in clinical samples of canine cataracts compared to normal lenses. Following exposure to UV, cultures of LEC significantly (p<0.05) increased expression of pAkt and telomerase activity. Treatment with AR-12 for both 8 and 24 h following UV irradiation significantly (p<0.01) decreased pAkt expression. When UV-exposed LEC were allowed to recover in the presence of either 5.0 or 10.0 µM AR-12, there was a significant (p<0.05) decrease in telomerase activity. In the ex vivo model of PCO, within the region of the capsulorhexis, PCO inhibition was maximally achieved with 10 μM of AR-12. A significant decrease in LEC was noted on the posterior capsules containing 5.0, 7.5, and 10 μM AR-12 compared to the control capsules (p<0.01). Telomerase activity decreased in a dose-dependent manner. One week of treatment with 10 μM AR-12, immediately following capsule excision, was sufficient to inhibit PCO formation, while a delay in exposure to AR-12 after 1 week of media incubation alone did not prevent PCO formation.

**Conclusions:**

pAkt is known to have roles in cell survival, proliferation, and migration, and this study suggests its inhibition immediately following cataract surgery may be a useful approach to prevent PCO.

## Introduction

Phacoemulsification extracapsular cataract extraction with intraocular lens (IOL) implantation is the most common ophthalmic surgical procedure performed today in human and veterinary ophthalmology [1,2]. Though current cataract surgery with IOL placement carries a greater than 95% success rate, the most common long-term postoperative complication in both humans and dogs is posterior capsule opacification (PCO) [1,2]. Postoperatively, the primary response of the remaining lens epithelial cells (LEC) is to proliferate and undergo epithelial-mesenchymal transition (EMT), that is, change from normal cuboidal epithelial cells into spindle shaped fibroblast-like cells that expresses α-smooth muscle actin [3,4]. The average time to significant clinical PCO in humans is 26 months postoperatively, with a range of three months to five years [5]. In humans, PCO occurs in 30%–50% of all surgical patients up to 5 years post-operatively, depending on age, location, and type of intraocular implant, while the incidence of PCO in dogs is 100% by one year post-operatively [5-9].

Telomerase is a ribonucleoprotein complex that primarily maintains telomeres but can also heal broken chromosomes and has anti-apoptotic functions [10,11]. Telomerase is absent from most normal somatic cells, hence their finite proliferative potential, but is found in germline and stem cells, the majority of cancers, and LEC [12-18]. Telomerase activity is almost threefold higher in cataractous LEC and significantly higher in lens capsules with PCO than in normal LEC [18]. We have found that canine and human lens capsules with PCO formation have high telomerase activity which may contribute to lenticular EMT by giving the cells unlimited proliferative capability [19].

Breakdown in the blood:aqueous barrier following cataract surgery can increase the expression of growth factors and cytokines in the aqueous humor, promoting EMT [20]. Many of the mitogens found in the aqueous humor, such as transforming growth factor-β (TGF-β) and platelet-derived growth factor (PDGF), can induce lenticular EMT through the Akt pathway [21,22]. Previously, we have shown that phorphylated Akt (pAkt) interacts with and phosphorylates telomerase in canine LEC that have undergone EMT, but not in normal canine LEC [23,24]. It is possible that induction of pAkt results in increased lenticular telomerase activity, thus promoting PCO formation. Because telomerase activity is only minimally expressed within the eye [18,25], the use of acute pAkt inhibition in the lens capsule following cataract surgery to prevent telomerase activation may be a potential strategy to prevent or reduce PCO. The aim of this study was to assess expression of pAkt in LEC undergoing EMT and to determine the effect of inhibiting signal transduction through Akt on PCO formation in an ex vivo canine lens capsule explant model.

In this study, the pharmacological inhibition of pAkt signaling was achieved through the use of AR-12 [26] (formerly OSU-03012; Arno Therapeutics, Inc., Parsippany, NJ), a novel small molecule inhibitor of phosphoinositide-dependent protein kinase-1 (PDK-1), the immediate upstream activating kinase of Akt, that is currently being evaluated in Phase I clinical trials in cancer patients. To evaluate the effect of AR-12 on LEC, primary cultures were stressed with ultraviolet (UV) irradiation and allowed to recover in the presence or absence of AR-12 before pAkt expression and telomerase activity were assessed [27]. To evaluate the effect of AR-12 in a PCO model, telomerase activity was measured and immunoreactivity of pAkt and telomerase reverse transcriptase (TERT) were determined as their expression have been shown to correlate with telomerase activity [19,28-30]. Integrin-linked kinase (ILK), α-smooth muscle actin (αSMA), and proliferating cell nuclear antigen (PCNA) were assessed as indicators of EMT, cell migration, and proliferation of LEC, respectively [4,31,32]. Expression of cleaved caspase-3 was used to evaluate whether cells were undergoing apoptosis in our ex vivo PCO model [33].

## Methods

### Samples

Normal eyes were obtained by enucleation from dogs in good general health that were humanely euthanized at a local animal shelter for population control purposes. The eyes were examined with a focal light source and deemed free of corneal disease, cataract, or other anterior segment disease. All dogs used in this study were estimated to be between 1 and 8 years of age, based on dentition and thickness of the anterior lens capsule. Globes were collected within one hour of death and placed in dilute betadine solution, then rinsed and immersed in 1× phosphate buffered saline solution (PBS, pH 7.2) until dissection. Excess conjunctiva and Tenon’s capsule were excised from the perilimbal region, and a stab incision was made into the anterior chamber 1 mm posterior to the limbus. Anterior lens capsules with adherent LEC were excised from each lens for protein extraction and immediately frozen and stored at −70 °C until extraction. Whole lenses and anterior lens capsules with adherent LEC were also fixed in 10% neutral-buffered formalin for immunohistochemical staining.

Cataractous lens capsules were obtained from clinically normal dogs undergoing elective phacoemulsification cataract surgery at The Ohio State University College of Veterinary Medicine. These capsules consisted of mature and hypermature cataracts that were considered either diabetic or non-diabetic in their etiology. These canine cataracts almost universally have LEC that have undergone EMT and posterior migration onto the posterior capsule [32]. Samples chosen for this study had obvious subcapsular plaques or diffuse LEC proliferation onto the posterior lens, evident at the time of surgery. Patients with only nuclear cataract were not included in this study. Cataractous samples were obtained by continuous curvilinear capsulorhexis and either immediately placed in 10% neutral-buffered formalin or snap frozen and stored at −70 °C until protein extraction.

All fixed samples were paraffin embedded, sections, stained with hematoxyline and eosin (H&E), and then examined by light microscopy. For immunohistochemical staining, samples were sectioned onto charged slides (ProbeOn Plus; Fisher Scientific, Pittsburg, PA).

### Primary cultures

Anterior lens capsules with adherent LEC were obtained via dissection from normal canine eyes, as described above. Following dissection capsules were incubated in trypsin (0.25% trypsin and 1× EDTA; Gibco, Carlsbad, CA) for 5 min at 37 °C. After incubation, the solution and lens capsule were centrifuged for 2 min at 0.3× g. Fluid was decanted and supplemented DMEM (containing 10% fetal bovine serum and 1% antibiotic/antimycotic [Gibco]) was then added. The solution, including the lens capsule, was transferred to a laminin-coated culture flask (Beckton-Dickinson, Franklin Lakes, NJ) an incubated in a humidified incubator at 37 °C and 5% CO_2_. LEC were grown until 90% confluence before re-plating.

### Irradiation and treatment with AR-12

Cultures of primary LEC were washed and covered with PBS, then exposed to UV irradiation. The UV source was from Philips TL20 W/12 RS UV-B lamps (American Ultraviolet Company, Murray Hill, NJ). These lights emit wavelengths of light between 280 and 400 nm, with a peak at 313 nm. The lamps emit approximately 60% UV-B and 40% UV-A [34]. Radiation was filtered through Kodacel (Eastman Kodak, Rochester, NY), to remove wavelengths below 280 nm [35]. Based on preliminary and previously published data, the cells were irradiated with either 0 or 600 J/m^2^, which can induce expression of both pAkt and telomerase [23,27]. Following exposure, PBS was removed and serum-free DMEM containing AR-12 was placed on the cells. The doses of AR-12 used were 0 (vehicle only), 2.5, 5.0, and 10.0 µM (n=6). Cells were collected 8 or 24 h after irradiation for immunoblot and telomerase activity analysis.

### Ex vivo capsules

An extracapsular cataract extraction with lens capsule dissection was performed as described previously [36-38]. Residual cortical material was removed gently by manual irrigation/aspiration with a coaxial cannula, but no effort was made to polish the anterior or posterior lens capsule. The lens capsule was excised from the zonular and vitreal attachments and pinned to a polymethylmethacrylate (PMMA) Petri dish also as previously described [38]. All capsules were cultured in a total of 5.0 ml of DMEM containing 1% antibiotic and antimycotic (Gibco).

### Treatment groups

Each group contained 8 lens capsules and 5 groups were treated with 0.0, 2.5, 5.0, 7.5, or 10.0 μM AR-12, which was synthesized in-house as described previously [26]. The lens capsules were incubated at 37 °C in 5% CO_2_, and the culture medium replaced every 3 days for 14 days. Morphologic observations were made with phase-contrast microscopy. Two additional groups of lens capsules were harvested (n=6 per group). One group was exposed to DMEM supplemented with 10.0 μM AR-12 for the first week of incubation, followed by a week of DMEM alone. The second group was exposed to DMEM alone for the first week of incubation, followed by DMEM supplemented with 10.0 μM AR-12 for the second week of incubation. After 2 weeks, all lens capsules were fixed in 10% neutral-buffered formalin, embedded in paraffin, and sectioned.

### Telomeric repeat amplification protocol (TRAP)

The TRAP assay, modified by the Intergen Company (Norcross, GA), is an extension of the original method [18]. In the first step, telomerase, if present in the protein lysate, adds several telomeric repeats onto the 3′ end of a biotinylated telomerase substrate oligonucleotide (b-TS). In the second step, the extended products are amplified by PCR using Taq polymerase, the b-TS and RP (reverse) primers, and a deoxynucleotide mix containing dCTP labeled with dinitrophenol, generating a ladder of products with six base increments, starting at 50 nucleotides (Chemicon, Temecula, CA). Briefly, for our studies, a master mix was prepared with 5× TRAP Reaction mix, Taq polymerase (Panvera Takara, Carlsbad, CA), and RNase-free water. One µg of protein was added to the Master mix for a final volume of 50 µl; all set-up steps were performed on ice. Primer extension was then performed at 30 °C for 30 min, followed by 33 cycles of amplification as follows: denaturation for 30 s at 94 °C and annealing for 30 s at 53 °C. Samples were held at 4 °C until detection using hybridization and ELISA (absorbance measured at 450 nm with reference wavelength of 690 nm) following manufacturer’s protocol, and electrophoresis on a 10% nondenaturing polyacrylamide gel that was subsequently stained with SYBR gold (1:10,000; Molecular Probes, Carlsbad, CA). By comparing the PAGE gel method of evaluating TRAP results with the ELISA methods, we have set 0.100 units as the lowest amount of detectable telomerase activity.

### Histological and immunohistological evaluation

Routine H&E staining was performed on ex vivo capsules to evaluate cell morphology and cell counts. All LEC adhered to the capsule were manually counted in 10 H&E sections per capsule. The quantitative analysis was performed on sections taken as close as possible to the capsule midline. Cell counts were performed by two independent observers.

Immunohistochemical staining was performed as previously described [38] using antibodies against TERT (1:350; Calbiochem, San Diego CA), pAkt (1:350; Santa Cruz, Santa Cruz, CA), cleaved caspase-3 (1:20; Oncogene, San Diego CA), ILK (1:200; Stressgen, San Diego CA), α-SMA (1:100; Spring Bioscience, Freemont CA), and PCNA (1:200; DAKO Corp., Carpinteria CA). Secondary antibodies included, as appropriate, anti-goat (Vector Laboratories, Burlingame CA), anti-rabbit (Biogenex Super Sensitive Link; Biogenex, San Ramon CA), and anti-mouse (Vector Laboratories) antibodies. DAKO Antibody Diluent with Background Reducing Components (DAKO) was used to dilute antibodies. Immunostaining was visualized with either diaminobenzidine tetrahydrochloride (DAB, DAKO) applied for 3 min at room temperature in the dark or with Vector NovaRED (Vector Laboratories) applied for 5 min at room temperature.

Appropriate positive and negative controls were performed with each experiment. Canine squamous cell carcinomas were used as a positive control for TERT, pAkt, α-SMA, ILK, and caspase-3. A normal canine lymph node was used as a positive control for PCNA. Negative controls omitted the primary antibody.

### Immunobloting

Whole cell protein was extracted from frozen positive controls, normal canine anterior lens capsules, and anterior capsulotomy samples from clinical canine cataracts using 1× CHAPs lysis buffer (Chemicon, Temecula, CA) in accordance with the manufacturer’s instructions with minor adaptations for our tissue as previously described [19]. Protein concentration was quantified using the Bradford protein assay (Bio-Rad, Hercules, CA).

Samples were denatured (95 °C, 3 min) in modified sodium dodecyl sulfate (SDS) sample loading buffer, and protein (15 µg) was separated by SDS/PAGE (10% acrylamide, v/v) at 150 V for approximately 1.5 h. After electrophoresis, proteins were transferred to a nitrocellulose membrane at 300 mA for 1.5 h. Nonspecific binding was blocked by incubating the membrane for 5 h at room temperature in 5% nonfat dry milk diluted in 1× Tris-buffered saline containing 0.1% Tween-20 (TBST), After blocking, membranes were incubated overnight with pAkt (Cell Signaling, Danvers, MA), diluted 1:500 in blocking buffer. The membrane was then washed extensively with 1× TBST, and secondary antibody (donkey anti-rabbit, Santa Cruz) was added in blocking buffer (1:8,000) for 1 h at room temperature. Protein was detected using the Pierce (Rockford, IL) Femto Immunoblotting system. Membranes were stripped (Pierce) and the technique was repeated using anti-β-actin antibody (1:5,000; Sigma-Aldrich, St. Louis, MO). Kodak 1D Image Analysis Software (Kodak Molecular Imaging, New Haven, CT) was used to obtain densitometry readings for all western blots.

### Statistical analysis

All statistical analysis was performed with Prism version 4.0 software (GraphPad Software Inc. San Diego, CA). All data was analyzed using one-way ANOVA (ANOVA) with Tukey’s post-test. The capsule cell counts were evaluated with a non-paired Students’ *t*-test. The level for statistical significance was set at p<0.05 for all comparisons. All graphs were generated in Prism version 4 and display the mean and standard error of the mean (SEM), represented by error bars.

## Results

### pAkt expression in normal and cataractous lens epithelial cells

**Figure 1:**
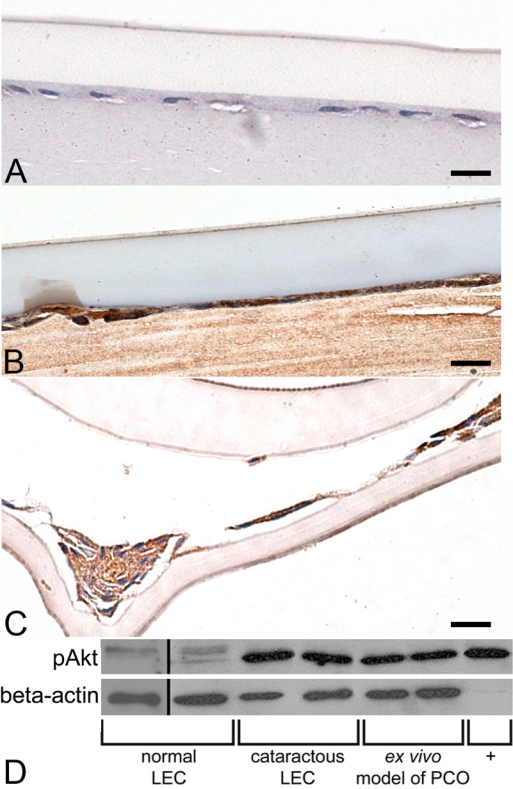
Expression of pAkt in normal canine LEC and in LEC undergoing EMT. **A**: In normal LEC there is little to no immunoreactivity, while LEC in **B**: clinical cataracts and **C**: clinical samples of PCO, show marked cytoplasmic staining for pAkt. The scale bar is equal to 30 µm. **D**: western blot analysis demonstrates increased expression of pAkt protein in both cataractous LEC and LEC in the ex vivo model of PCO formation, compared to normal LEC. The ‘+’ indicates a canine mast cell tumor which was used as a positive control for pAkt expression.

As evaluated by immunohistochemisty, normal canine LEC had minimal to no detectable pAkt protein present (Figure 1A), while immunostaining for pAkt was strong predominantly in the cytoplasm of naturally occurring cataractous canine LEC (Figure 1B) and clinical samples of canine PCO (Figure 1C). Western immunoblotting confirmed that LEC from clinical cataract samples had increased expression of pAkt protein compared to normal LEC (Figure 1D). Samples from our ex vivo model of PCO formation demonstrated strong expression of pAkt, as observed by western blot (Figure 1D).

### Expression of pAkt and telomerase activity follow irradiation and AR-12 treatment

**Figure 2:**
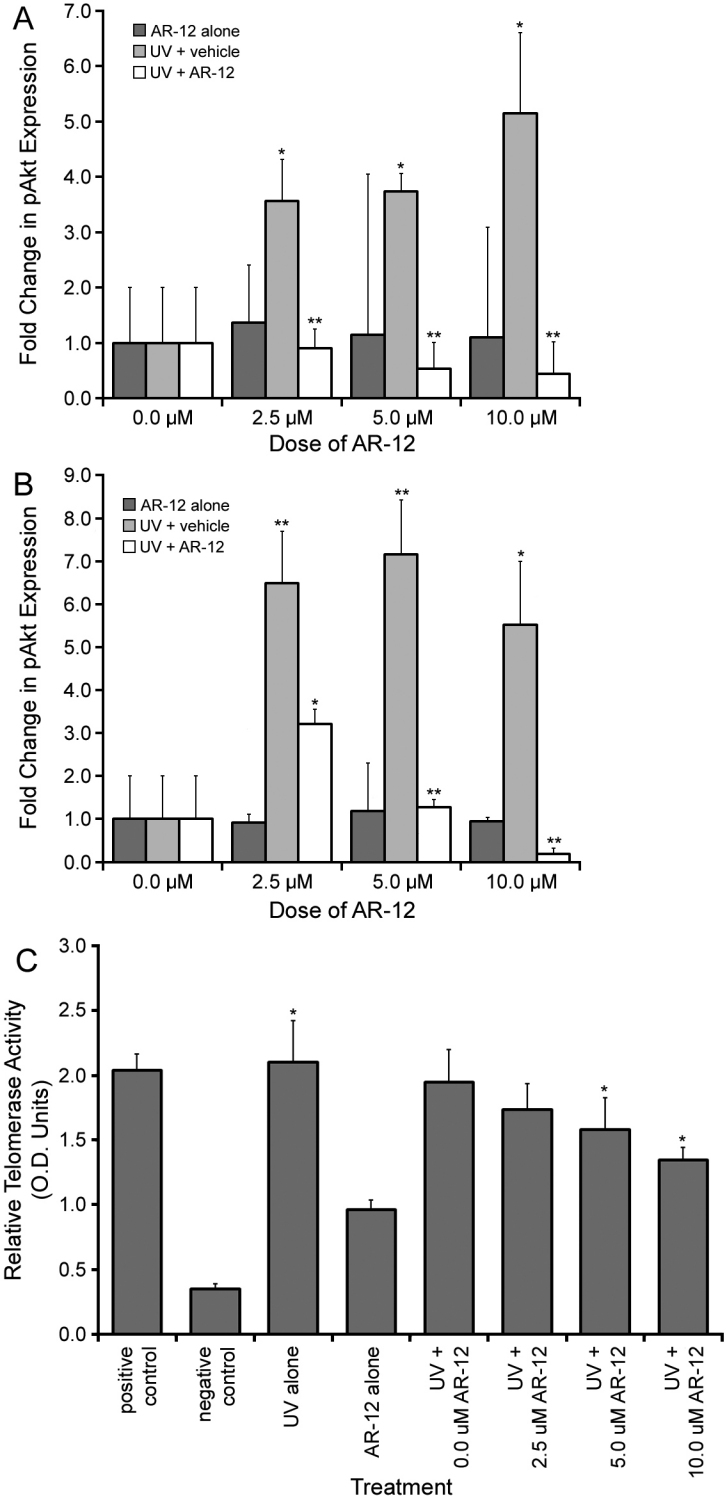
Expression of pAkt and telomerase activity following UV irradiation and AR-12 treatment. **A**: pAkt expression in LEC 8 h after treatment with AR-12 alone, UV and vehicle, or UV and AR-12. There was a significant increase in LEC pAkt protein expression following UV irradiation in the absence of AR-12 (light gray bars). A significant decrease in pAkt levels was found in LEC that were irradiated and allowed to recover in the presence of AR-12 (white bars), compared to LEC that were irradiated and allowed to recover in the absence of AR-12. Treatment with AR-12 alone did not impact the expression of pAkt (dark gray bars). **B**: pAkt expression in LEC 24 h after treatment with AR-12 alone, UV and vehicle, or UV and AR-12. There was a significant fold change in pAkt expression in LEC that were irradiated and allowed to recover in the absence of AR-12 (light gray bars). pAkt expression was significantly reduced when LEC were allowed to recover from irradiation in the presence of AR-12 (white bars). **C**: Telomerase activity in LEC 24 h after treatment. Telomerase activity was significantly increased in LEC following UV exposure. A significant decrease in telomerase activity was noted in LEC that were irradiated and allowed to recover in the presence of 5.0 or 10.0 µM AR-12 for 24 h. Treatment with AR-12 alone did not alter telomerase activity in LEC (* indicates p<0.05 and ** indicates p<0.01).

Irradiation of LEC significantly induced expression of pAkt at both 8 (p<0.05; Figure 2A) and 24 (p<0.01; Figure 2B) h compared to controls, as observed by western blot analysis. In the presence of AR-12, all doses tested were capable of significantly (p<0.01) decreasing pAkt expression in irradiated LEC compared to LEC that received UV irradiation in the absence of AR-12 (Figure 2A,B). This was observed at both 8 (Figure 2A) and 24 (Figure 2B) h. Cells treated with AR-12 alone did not significantly alter pAkt expression (Figure 2A,). As previously demonstrated [27], when assessed by the TRAP assay, telomerase activity was significantly (p<0.05) increased in LEC that were exposed to irradiation and allowed to recover for 24 h (Figure 2C). When UV-exposed LEC were allowed to recover in the presence of either 5.0 or 10.0 µM AR-12, there was a significant (p<0.05) decrease in telomerase activity compared to the cells that were irradiated and received 0.0 µM AR-12 (Figure 2C).

### Control ex vivo lens capsules

**Figure 3:**
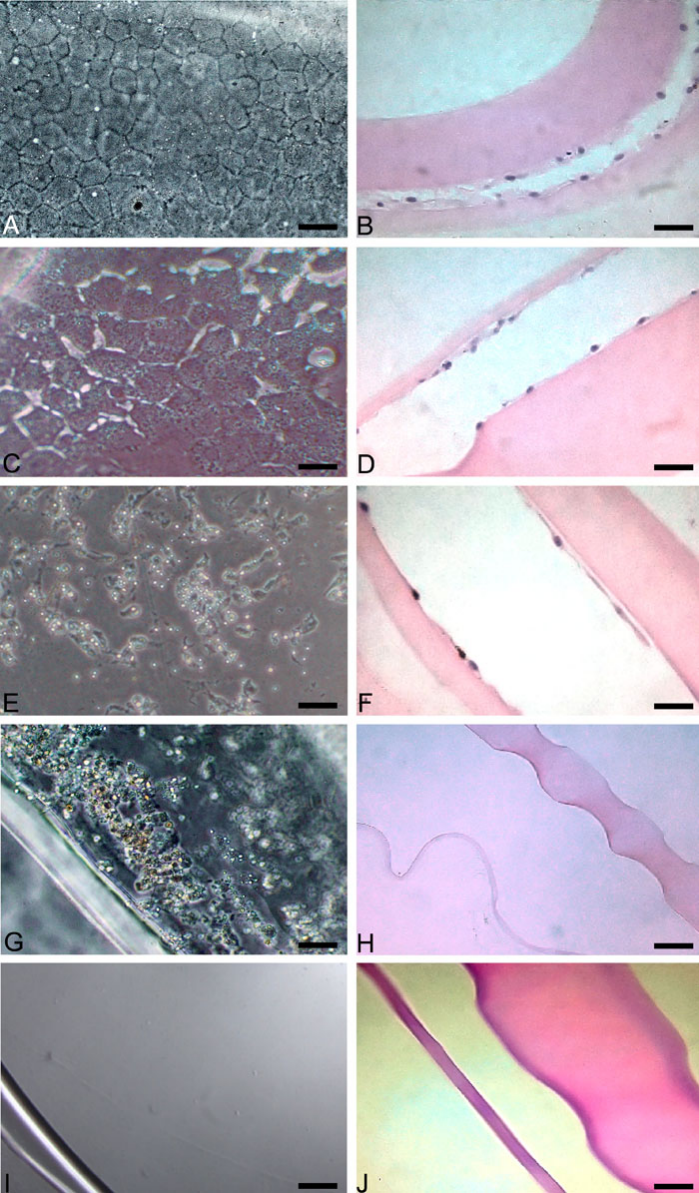
AR-12 treatment in the ex vivo model of PCO following 14 days. **A**: Lens capsules treated with DMEM media and vehicle as viewed by phase contrast microscopy (scale bar is equal to 20 µm); the cells are healthy and cuboidal to hexagonal in shape with very little anisocytosis. **B**: Lens capsules treated with DMEM and vehicle (H&E, scale bar is equal to 30 µm); LEC are of normal morphology and present across the anterior and posterior lens capsules. **C**: Lens capsules treated with 2.5µM AR-12 and viewed by phase contrast microscopy (scale bar is equal to 20 µm); the cells are mostly cuboidal to hexagonal in shape but some vacuolation is present. **D**: Lens capsules treated with 2.5 µM AR-12 (H&E, scale bar is equal to 30 µm); LEC display anisocytosis and are fewer in number compared to control. **E**: Phase contrast photograph (scale bar is equal to 20 µm) or **F:** H&E sectioning (scale bar is equal to 30 µm) of lens capsules treated with 5 µM AR-12; there is marked vacuolation, cells lack normal morphology and there are areas of capsule devoid of epithelial cells. Lens capsules treated with 7.5 µM AR-12 as viewed by either **G**: phase contrast microscopy (scale bar is equal to 20 µm) or **H**: H&E sectioning (scale bar is equal to 50 µm) had no normal LEC; few cells and cellular debris remains within the lens capsule and are adherent to the anterior or posterior lens capsules. **I**: Lens capsules treated with 10 µM AR-12 and viewed by phase contrast microscopy (scale bar is equal to 20 µm); the lens capsule is devoid of LEC. **J**: Lens capsules treated with 10 µM AR-12 (H&E, scale bar is equal to 30 µm); anterior and posterior lens capsules are without LEC.

Immediately post-dissection, lens capsules had visible LEC present on the anterior capsule only. By 3 days post-dissection, untreated LEC had migrated across the anterior and posterior lens capsules. By 7 days post-dissection, LEC were present on the posterior capsule in the central visual axis in most lens capsules, and all had reached confluence by 14 days post-extraction (Figure 3A). The LEC morphology was cuboidal to hexagonal and there was little anisocytosis. Hematoxylin and eosin staining confirmed the phase contrast findings (Figure 3B). At 2 weeks post-dissection, the control lens capsules contained confluent cells on both the anterior and posterior capsules with focal areas of LEC hyperplasia in the equatorial region.

### AR-12 treated ex vivo lens capsules

All LECs in the lens capsules treated with 2.5 μM AR-12 in DMEM displayed mild intracellular vacuolization and anisocytosis. Some areas of lens capsule contained sparse LEC, but confluence was visible by 7 days after sham cataract surgery. Histopathologically, LEC were flattened and visibly fewer in numbers compared to control lens capsules (Figure 3C,D).

All lens capsules treated with 5 μM of AR-12 in DMEM had areas devoid of LECs and confluence was not achieved by 14 days after sham cataract surgery. LEC were severely vacuolated and varied in size and shape. Histologically, most areas of the anterior and posterior lens capsules were devoid of LEC; cells, as observed with phase contrast, indicated abnormal morphology and vacuolization (Figure 3E,F).

Neither lens capsule groups treated with 7.5 nor 10 μM AR-12 in DMEM supported LEC migration and proliferation. The lens capsules contained minimal numbers of LEC on phase contrast evaluation of 7.5 μM-treated capsules, and capsules were devoid of all LEC on phase contrast evaluation of 10 μM AR-12-treated. These findings were confirmed with histopathology (Figure 3G-J).

### Effects of late Akt inhibition on LEC

Lens capsules treated with DMEM only for 7 days, followed by 7 days of treatment with 10 μM AR-12 in DMEM were grossly and histologically similar to the control lens capsules. By 7 days post dissection, confluence was attained across the entire posterior capsule in most lens capsules (data not shown).

### Effects of early Akt inhibition of LEC

Lens capsules treated with 10 μM AR-12 in DMEM for 7 days, then treated with compound-free media for 7 days were grossly and histologically similar to those lens capsules treated with 10 μM AR-12 continuously for 14 days. The lens capsules were devoid of all LEC on phase contrast evaluation (data not shown).

### Telomerase activity

**Figure 4:**
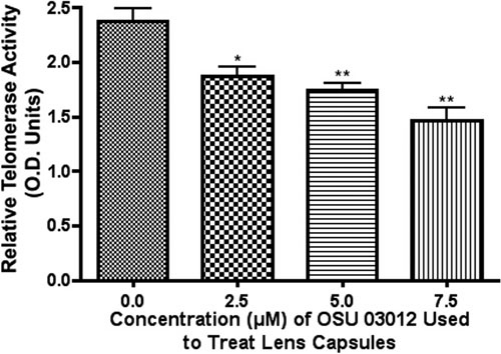
Comparative analysis of telomerase activities in canine lens capsules treated with various doses of AR-12. There is a significant decrease in activity in all of the treatment groups compared to the controls. (* indicates a p<0.05; ** indicates a p<0.01).

All capsules with LEC present underwent TRAP evaluation for the presence or absence of telomerase activity. There was a significant decrease in telomerase activity in the AR-12 treated groups compared to the untreated controls (Figure 4). Capsules in the 10 µM treatment group did not undergo TRAP evaluation because there were no LEC were present following 14 days of culture.

### Lens epithelial cell counts

**Figure 5:**
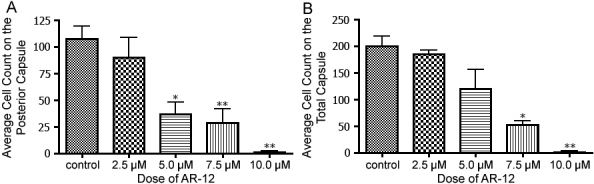
Posterior and total lens capsule cell counts. **A**: The number of LEC present on the posterior capsule was significantly lower in the groups treated with 5.0, 7.5, and 10.0 uM AR-12 than in control capsules (* is p<0.05; ** is p<0.01). **B**: The total number of LEC present within the entire capsule was significantly lower in capsules treated with 7.5 and 10.0 uM AR-12 compared to the control capsules (* is p<0.01; ** is p<0.001).

The posterior capsule LEC counts of the 5.0, 7.5, and 10.0 uM -treated capsules were significantly lower than those of the control capsules (p<0.05 and p<0.01; Figure 5A). Similar results were obtained by two independent observers. The complete capsule LEC count of the 5.0 uM AR-12 was lower than those of the control capsules, however, this was not a significant difference (Figure 5B). The complete capsule LEC count of the 7.5 and 10.0 uM treated capsules were significantly lower than the complete control capsule LEC counts (p<0.01 and p<0.001; Figure 5B).

### Immunohistochemical staining following pAkt inhibition

**Figure 6:**
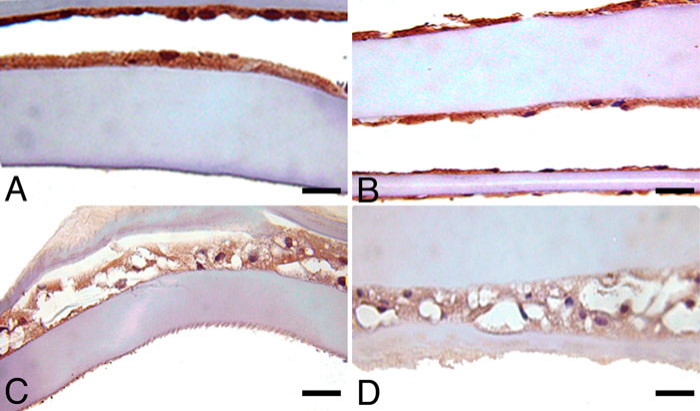
Immunohistochemistry for pAkt in canine lens capsules treated with AR-12 for 14 days. **A**: Untreated control capsules had strong expression of pAkt; as the concentration of AR-12 increased, the expression of pAkt decreased. **B**: 2.5 µM AR-12; **C**: 5.0 µM AR-12; **D**: 7.5 µM AR-12. Scale bar in all images is equal to 30 µm.

**Figure 7:**
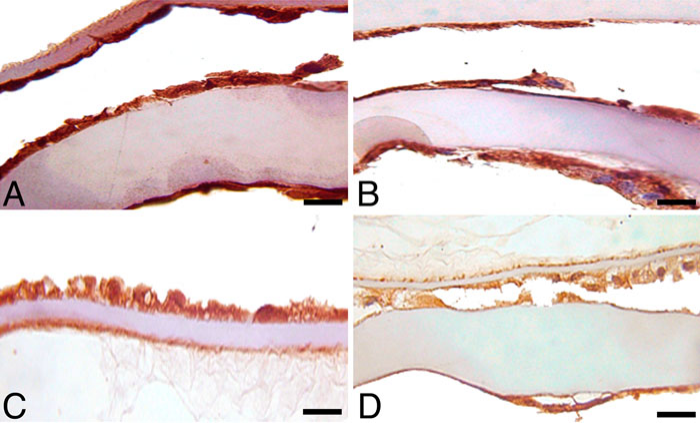
Immunohistochemistry for TERT in canine lens capsules treated with AR-12 for 14 days. **A**: Untreated control capsules had strong expression of TERT; as the concentration of AR-12 increased, the expression of TERT decreased. **B**: 2.5 µM AR-12; **C**: 5.0 µM AR-12; **D**: 7.5 µM AR-12. Scale bar in all images is equal to 30 µm.

**Figure 8:**
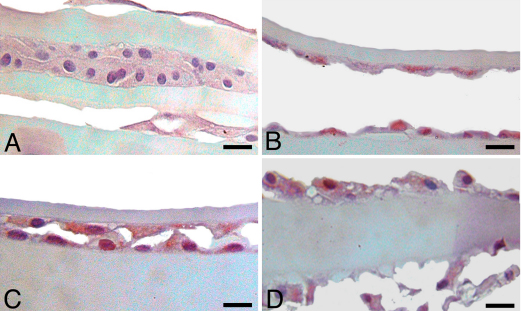
Immunohistochemistry for cleaved caspase-3 in canine lens capsules treated with AR-12 for 14 days. **A**: Untreated control capsules had no cells positive for caspase-3; as the concentration of AR-12 increased, the number of caspase-3 positive cells increased. **B**: 2.5 µM AR-12; **C**: 5.0 µM AR-12; **D**: 7.5 µM AR-12. Scale bar in all images is equal to 30 µm.

Immunohistochemical staining of LEC in all lens capsules, in all treatment groups, excluding 10 µM due to a lack of cells, showed equivalent moderate immunoreactivity for ILK, α-SMA, and PCNA (data not shown). There was positive immunoreactivity for TERT and pAkt in all treatment groups, but a subjective decrease in staining was observed in the 7.5 μM-treated groups, in comparison to untreated control, 2.5 and 5 μM-treated groups (Figure 6 and Figure 7). Caspase-3 immunoreactivity was variable, but increased numbers of positive cells were seen in lens capsules treated with AR-12 concentrations at 5 μM and above (Figure 8).

## Discussion

PCO is the most common post-operative complication of human and canine cataract surgery [1,2,5-9]. Postoperatively, the primary response of the remaining LEC is to undergo EMT, proliferate, and increase production of aberrant extracellular matrix proteins. Additional morphological alterations in these LEC include cell elongation, organelle loss, and nucleolar chromatin condensation [39]. It has been shown that the anterior LECs in both rabbits and humans initially undergo hyperplasia and transform into spindle-shaped myofibroblasts [40,41] that, along with the LEC at the lens equator, migrate posteriorly and abnormally occupy the posterior capsule [5,42].

Multiple growth factors found within the aqueous humor have been documented to play important roles in the induction of PCO [20]. Evidence to implicate TGF-β in PCO formation is strong and supported by multiple studies; in fact, Wormstone et al. [22,43-45] found that acute exposure to TGF-β could produce long-term changes in lens capsules. There is substantial cross-talk between the TGF-β and PI3K/Akt pathway, particularly during EMT [22,46]. Other growth factors, such as PDGF, have previously been found to induce and regulate LEC proliferation and may be involved in modulation of cellular migration [47-50]. One recent study has found that induction of the PDGF pathway in human LEC resulted in a PI3K/Akt pathway dependent increase in cellular migration [21]. It is likely that aqueous humor growth factors such as TGF-β and PDGF, can induce expression of pAkt in LEC resulting in PCO, particularly if there is breakdown of the blood:aqueous barrier postoperatively. As such, the involvement of the PI3K/Akt pathway in the formation of PCO is potentially important in designing therapeutic strategies to inhibit the formation of PCO.

Several pharmacologic agents have been tested to inhibit PCO formation; however, to date, there are no reports on the use of specific Akt inhibitors to prevent LEC migration and proliferation. Research performed in our laboratory indicates that use of celecoxib an ex vivo model, results in decreased PCO formation [38]. Celecoxib is capable of inhibiting both COX-2 and Akt; it was speculated that one mechanism celecoxib was effective in preventing in vitro PCO formation was due to increased Akt regulated apoptosis [38]. In vitro and postoperative use of the NSAID, diclofenac, has been associated with decreased PCO formation [51-53]. Diclofenac enhanced apoptosis has been linked to Akt downregulation in other cell types and may exert similar effects in LEC [54]. Alternate pharmacologic agents with no known Akt inhibiting mechanisms have been used to prevent PCO formation in vitro. Some examples include, seleno-cystamine coated IOLs which have the potential to serve as an effective, longer lasting means of preventing PCO, although in vivo trials are needed [55]. Hypoosmolar and antimetabolites such as mitomycin, daunomycin, and 5-fluorouracil, have been effective in inhibiting PCO in vitro through LEC lysis [56-60]. Unfortunately, in vivo concentrations high enough to inhibit LEC proliferation, whether as a single application or as a sustained release, have resulted in toxic effects to the corneal endothelial cells, iris, ciliary body and/or retina [61].

AR-12 is a novel small molecule derived from the chemical backbone of the COX-2 inhibitor, celecoxib. It is devoid of COX-2 inhibitory activity [26]. In this study, we have shown that AR-12 can induce LEC death when used at various concentrations in vitro and can decrease PCO formation in the canine lens capsule explant model. There was a positive correlation between concentration of AR-12 used and LEC apoptosis, as determined by decreased cell viability and increased caspase-3 expression in non-confluent LEC. Because of this, we speculate that pAkt is critical to the initial adhesion, migration, or morphological change needed for LEC to undergo EMT. Additionally, this is based on the observation that exposure of the LEC for only one week with AR-12 induced apoptosis, while a 7-day delay in AR-12 treatment failed to induce observable changes in experimental PCO formation.

Shorter exposures of LEC to AR-12 were not tested to determine the minimal treatment durations needed to induce cell death. Normal cells that require adhesion to a basement membrane will die by an apoptotic mechanism termed anoikis shortly after they lose this adhesion unless they have undergone transformation or are neoplastic [62]. PI3K/Akt signaling is a critical pathway for anchorage-dependent cell survival and growth and protects cells from anoikis by inactivating key apoptotic proteins and enhancing anchorage-independent cell cycle progression [62]. Anoikis is a plausible mechanism by which LEC are dying in these experiments as the LEC are visibly separating from the lens capsule and dying.

Data presented here demonstrates that AR-12 can significantly inhibit telomerase activity in canine LEC; AR-12 also subjectively inhibited pAkt and TERT expression. We cannot be certain that this effect is not a reflection of cell loss, but equal protein concentrations are used in testing to minimize this variable. This finding suggests that pAkt and TERT may be necessary at an initial step for EMT to occur in LEC during PCO and cataract formation.

To evaluate cellular changes in this model, ILK, α-SMA, and PCNA were used as indicators of cell migration, EMT, and proliferation of LEC [4,31,32]. Immunohistochemistry for α-SMA and PCNA indicates that our ex vivo model accurately mimics the migration and proliferation of LEC seen during in vivo PCO formation. ILK can influence a wide range of cellular processes in development and disease (proliferation, migration, EMT) [4], and many of these effects may be due to its activation of Akt and interaction with the PI3K pathway. Transfection of LEC with ILK-expressing constructs induces a morphologic change that resembles EMT [4]. In support of this, LEC present in our ex vivo PCO model were undergoing EMT and were positive for ILK expression. In this study, we did not see any changes in expression of ILK in capsules that were treated with AR-12. These findings support the hypothesis that ILK is an upstream mediator of Akt activation and not directly affected by inhibition of pAkt.

In vivo studies using inhibitors of Akt signaling perioperatively to control PCO formation are planned in animal models. Previous use of chemical inhibitors of PCO have been successful in vitro but failed clinically due to their toxic effects on the corneal endothelium and other cell types. Other intraocular cell types, including the corneal endothelium, express pAkt, suggesting that its inhibition may cause corneal edema. However, preliminary data from our laboratory indicate that AR-12 is not toxic to corneal endothelial cells when cells were continually exposed by intracameral infusion for 10 min at a concentration of 20 µM as determined by cell morphology and the absence of caspase-3 staining. Therefore, we believe that the use of AR-12 intraoperatively or by impregnation of an IOL, may be safe and effective in inhibiting PCO. Further studies are in progress to evaluate the minimal exposure time necessary for effective PCO inhibition.
